# Esthetic, Periodontal, and Functional Outcomes of Orthodontic Space Closure Versus Prosthetic Replacement for Congenitally Missing Maxillary Lateral Incisors: A Systematic Review

**DOI:** 10.7759/cureus.101260

**Published:** 2026-01-10

**Authors:** Majed M Althubaiti, Duaa Y Nunu, Ali H Alshawi, Amani H Alqurashi, Manar K Al-Lashlash, Asem A Alnouman, Nada S Alrashidi, Waleed M Alrashdi, Saleh I AlWatpan, Khalid F Alshammari, Maram K Almutairi, Mohammed H Alalawi, Zakiah K Barri, Shahd S Alswail

**Affiliations:** 1 Periodontics, Ministry of National Guard, Jeddah, SAU; 2 General Dentistry, Najran Speciality Dental Center, Najran Health Cluster, Ministry of Health, Najran, SAU; 3 College of Dentistry, University of Hail, Hail, SAU; 4 General Dentistry, College of Dentistry, King Abdulaziz University, Jeddah, SAU; 5 General Dentistry, College of Dentistry, Imam Abdulrahman Bin Faisal University, Dammam, SAU; 6 General Dentistry, Security Force Hospital, Riyadh, SAU; 7 Collage of Dentistry, Qassim University, Qassim, SAU; 8 General Dentistry, College of Dentistry, University of Hail, Hail, SAU; 9 College of Dentistry, Taibah University, Al-Madinah Al-Munawwarah, SAU; 10 General Dentistry, Lora Dental Clinic, Jeddah, SAU; 11 General Dentistry, Qassim University, Qassim, SAU

**Keywords:** canine substitution, congenitally missing lateral incisors, maxillary lateral incisor agenesis, orthodontic space closure, prosthetic replacement

## Abstract

Congenital absence of the maxillary lateral incisors is a common developmental anomaly that may compromise smile esthetics, periodontal health, and occlusal function, and it is typically managed by either orthodontic space closure with canine substitution or space opening followed by definitive prosthetic replacement. This systematic review aimed to compare esthetic, periodontal, and functional/occlusal outcomes between these two treatment approaches. A systematic search of PubMed, the Cochrane Library, LILACS, and Semantic Scholar was conducted from inception to 4 December 2025 in accordance with PRISMA 2020. Eligible studies included comparative clinical and cross-sectional designs reporting at least one relevant outcome domain; no randomized controlled trials were identified. Screening and data management were performed using Rayyan, risk of bias was assessed using ROBINS-I, and certainty of evidence was evaluated using the GRADE approach; due to substantial heterogeneity in study designs and outcome measures, meta-analysis was not performed, and findings were synthesized narratively. The search identified 1,244 records; after removal of 284 duplicates, 960 records were screened, 113 full texts were assessed, and 19 studies were included (18 retrospective and one prospective). Fifteen studies assessed esthetic outcomes, seven evaluated periodontal outcomes, and six reported functional/occlusal outcomes, using combinations of clinical examinations, photographic assessments, questionnaires, and cast/model analyses. Overall, orthodontic space closure yielded esthetic outcomes that were frequently acceptable to patients and lay observers. Periodontal findings were generally comparable across approaches, with prosthetic restorations more often associated with localized plaque-retentive challenges, and functional/occlusal outcomes appeared broadly similar without consistent evidence of increased temporomandibular dysfunction attributable to either modality. Most studies demonstrated moderate to serious risk of bias, and the overall certainty of evidence was low. Within these limitations, both strategies appear clinically acceptable, while orthodontic space closure may offer favorable periodontal profiles and esthetic acceptability, particularly from the lay perspective, while avoiding certain long-term prosthetic-related complications, underscoring the need for higher-quality prospective comparative research.

## Introduction and background

Tooth agenesis refers to the developmental failure of one or more teeth and may present as complete absence (anodontia) or partial absence (partial anodontia). The most common form of tooth agenesis, hypodontia, involves the congenital absence of at least one permanent tooth [1]. Based on epidemiological statistics, hypodontia is relatively common globally. It is typically reported to occur within the range of approximately 2.8% to 11.3%, with varying prevalence estimates across different populations, age groups, and diagnostic approaches [2]. Genetic background, study design, and inclusion criteria are some factors that contribute to this wide variation in reported prevalence rates [2,3].

The maxillary lateral incisor is among the most frequently missing teeth in hypodontia, with reported prevalence commonly ranging from approximately 1.91% to 3.6% [3]. Across pooled estimates for missing teeth, maxillary lateral incisors are generally the most commonly absent teeth after third molars and mandibular second premolars [2,3]. Sex-based differences have also been identified, with females more frequently affected than males in several epidemiological studies [2,3]. Additionally, maxillary lateral incisor agenesis (MLIA) is frequently accompanied by other dental anomalies, such as microdontia, peg-shaped laterals, delayed eruption, transposition, or altered eruption paths. This indicates that MLIA is a part of a broader impairment in dental development [4-7].

Genetic and clinical studies have found that tooth agenesis can emerge from disruptions in odontogenesis pathways [5]. Accordingly, MLIA may occur as an isolated familial anomaly or in association with multisystem syndromic and craniofacial conditions. In non-syndromic cases, variations in genes involved in tooth development have been linked to agenesis phenotypes, and genetic susceptibility may influence the affected teeth and whether the absence is unilateral or bilateral [1,5]. Studies have also investigated the genetic risk factors for MLIA. The findings indicate that an inherited component is biologically plausible, reinforcing the need to treat MLIA not only as a local tooth absence but also as a developmental phenotype with broader dental implications [6].

Clinically, MLIA is highly relevant because it affects the anterior esthetic zone and has significant esthetic and functional consequences. The absence of a maxillary lateral incisor can lead to diastemas, midline discrepancies, mesial migration of canines, and drifting or rotation of central incisors, often complicating alignment and smile harmony [4]. Furthermore, as the lateral incisor contributes significantly to anterior tooth proportion and smile balance, even small changes in width relationships can meaningfully influence smile esthetics perceived by professionals and lay observers [8-10]. These esthetic changes may cause a psychosocial burden, particularly among patients with high smile lines or strong esthetic expectations, rendering treatment planning more than a purely mechanical space-management decision [4].

From a functional perspective, MLIA management can influence anterior guidance and occlusal schemes. Anterior guidance typically involves canine guidance and group function, and clinical decisions that move canines into lateral incisor positions (canine substitution) may modify guidance patterns depending on final tooth positioning and morphology [8]. Occlusal scheme is also biologically applicable because differences in guidance and contacts can influence elevator muscle activity, which supports functional harmony when MLIA is treated by either space closure or prosthetic replacement [9]. Accordingly, the outcomes of MLIA therapy should be evaluated not only by esthetic appearance but also by periodontal stability and functional adaptation over time [8,9].

The management of MLIA depends on patient-specific factors, including age, dental and skeletal malocclusion, facial profile, smile line, and the size, shape, and color of the canines, as well as periodontal biotype and anticipated long-term stability [4]. Treatment options broadly include orthodontic space closure with canine substitution or orthodontic space opening followed by prosthetic replacement [11-14]. To improve lateral-incisor simulation and optimize gingival architecture in space closure strategies, interdisciplinary finishing is typically required, such as enamel recontouring, additive composite build-ups, reshaping, or bleaching [12]. Prosthetic replacement selection is influenced by age, growth completion, occlusal demands, and restorative risk profile, and the options include resin-bonded bridges, conventional fixed partial dentures, removable prostheses, and single-tooth implants [13-16]. Importantly, recent studies emphasize that despite their predictability, implants may present long-term esthetic and biologic limitations in the anterior maxilla. Conversely, space-closure approaches may avoid prosthetic-related risks but introduce esthetic trade-offs related to canine morphology and gingival zenith [15,16].

Although clinical guidance on MLIA treatment planning is well described, the comparative evidence base remains limited because studies often differ in follow-up duration, outcome definitions, and assessment methods. In addition, the desired outcomes may vary depending on whether the focus is periodontal health, esthetic integration, or functional performance [4,11-16]. As a result, treatment planning is frequently influenced by practitioner preference, patient expectations, and local feasibility rather than a consistently synthesized comparative outcome framework [4,11-16]. Therefore, this systematic review aims to critically evaluate and summarize the available evidence comparing orthodontic space closure and prosthetic replacement in patients with congenitally missing maxillary lateral incisors, focusing on periodontal, esthetic, and functional outcomes.

Overall, the clinical decision between orthodontic space closure and prosthetic replacement remains dependent on balancing three outcome domains: periodontal stability, esthetic integration, and long-term functional performance. However, the comparative evidence is scattered across heterogeneous study designs and outcome measures. To address this gap, this systematic review was designed to answer the following question: In patients with congenitally missing maxillary lateral incisors, what are the periodontal, esthetic, and functional outcomes of orthodontic space closure compared with prosthetic tooth replacement?

## Review

Methodology

This systematic review was conducted in accordance with the PRISMA 2020 guidelines to ensure consistency and reproducibility [17].

Eligibility Criteria

The Population-Interventions-Comparisons-Outcome-Study design (PICOS) strategy was adopted. Studies were included if they met the following criteria: Population (P): Patients with congenitally missing maxillary lateral incisors were included. Only non-syndromic individuals with agenesis confirmed clinically and radiographically were eligible. Patients with traumatic loss of lateral incisors or caries-related tooth loss were excluded. Intervention (I): Orthodontic space closure, performed typically with canine substitution and optional reshaping or bleaching, was the intervention of interest. Comparison (C): Tooth replacement approaches, including single implants, resin-bonded bridges (RBB/Maryland), conventional fixed partial dentures, or other definitive prosthetic restorations, were eligible comparators. Studies using ideal dentition control groups or digitally modified images representing ideal esthetic conditions were included only as supplementary evidence for esthetic perception. They were not used to support comparative clinical conclusions between orthodontic space closure and prosthetic replacement. Outcome (O): Eligible studies were required to report at least one of the following as primary outcomes: periodontal outcomes, esthetic outcomes, or functional outcomes related to restoration or treatment stability. Study Design (S): Randomized controlled trials (RCTs), non-randomized controlled trials, and comparative study designs, including prospective or retrospective cohorts and cross-sectional comparative studies, were included.

PICOS Framework

*​​​​​​​*A detailed summary of the Population, Intervention, Comparison, Outcomes, and Study design (PICOS) eligibility criteria applied in this systematic review is presented in Table 1. The table summarizes both inclusion and exclusion criteria used for transparent study selection.

**Table 1:** PICOS framework for the systematic review

Inclusion Criteria	Exclusion Criteria
Population (P)	
Patients with congenitally missing maxillary lateral incisors. Unilateral or bilateral missing teeth. Adolescents or adults of both sexes. Non-syndromic individuals.	Syndromic patients (e.g., cleft lip/palate, ectodermal dysplasia, etc) Patients with traumatic or caries-related tooth loss instead of congenital absence.
Intervention	
Orthodontic space closure: Canine substitution; Canine reshaping/ composite/ bleaching.	Orthognathic surgery
Comparison	
Prosthetic space opening, including single-tooth implant; resin-bonded bridge (RBB/Maryland); conventional fixed partial denture; removable prosthesis; ideal dentition control groups, or digitally modified images representing ideal esthetic conditions.	Prosthesis for multiple missing teeth other than lateral incisors. Temporary prosthesis. Removable appliances only for retention purposes.
Outcome	
Studies must report at least one of the following. Primary Outcomes: Periodontal outcomes - Probing depth, gingival height, attachment level, papilla height, bone levels. Esthetic outcomes - Smile esthetics, gingival zenith, patient satisfaction, tooth proportion, color match. Functional outcomes - TMD Secondary Outcomes: Occlusal outcomes Patient-reported outcomes	Skeletal/dental cephalometric readings
Study Design	
Randomized controlled trails (RCTs), non-randomized comparative studies, prospective or retrospective cohort studies, case-control or cross-sectional studies.	Case reports, case series, narrative reviews, systematic reviews, expert opinions.

Information Sources and Search Strategy

The electronic databases PubMed, Cochrane Library, LILACS, and Semantic Scholar were systematically searched from inception to 4 December 2025. A combination of Medical Subject Headings (MeSH) terms and keywords related to lateral incisor agenesis, orthodontic space closure, and prosthetic replacement was used. Search strategies were adapted for each database. No filters were applied in any database, except for LILACS, where an English language filter was used due to feasibility. All other databases were searched without any language limits. The detailed search strings used in this review are presented in the search strategy provided in Table 2.

**Table 2:** Detailed search strategies across bibliographic databases

Database	Search Interface	Search String	Filters Applied	No. of Hits
PubMed	Advanced search builder	("Tooth Abnormalities"[MeSH] OR "Hypodontia"[MeSH] OR hypodontia[Title/Abstract] OR "congenitally missing lateral incisor"[Title/Abstract] OR "lateral incisor agenesis"[Title/Abstract] OR "maxillary lateral incisor"[Title/Abstract]) AND ("Orthodontic Space Closure"[MeSH] OR "Orthodontics"[MeSH] OR "canine substitution"[Title/Abstract] OR "space closure"[Title/Abstract]) AND ("Dental Implants"[MeSH] OR "Prosthodontics"[MeSH] OR "Prosthesis Design"[MeSH] OR implant*[Title/Abstract] OR "prosthetic replacement"[Title/Abstract] OR "resin bonded bridge"[Title/Abstract] OR "RBB"[Title/Abstract] OR "fixed partial denture"[Title/Abstract] OR bridge[Title/Abstract]) AND ("Periodontal Index"[MeSH] OR "Esthetics, Dental"[MeSH] OR "Dental Restoration, Permanent"[MeSH] OR periodontal[Title/Abstract] OR esthetic[Title/Abstract] OR aesthetic[Title/Abstract] OR restorative[Title/Abstract])	None	424
Cochrane Library	Search Manager	("lateral incisor" OR "lateral incisor agenesis" OR "missing lateral incisor") AND (orthodontic OR "space closure" OR "canine substitution") OR ("lateral incisor" OR "lateral incisor agenesis" OR "missing lateral incisor") AND (prosthetic OR prosthodontic OR implant OR "resin bonded bridge" OR "fixed partial denture")	None	360
Semantic Scholar	Basic keyword search	("lateral incisor" AND ("canine substitution" OR implant) AND periodontal)	None	408
LILACS	Basic keyword search	("lateral incisor" OR "lateral incisor agenesis" OR "missing lateral incisor" OR hypodontia) AND ("space closure" OR "canine substitution" OR orthodontic) AND (implant OR prosthetic OR prosthodontic OR "resin bonded bridge" OR "fixed partial denture" OR bridge)	English Language	52

Data Management and Screening

For data management and screening, all records retrieved from the database searches were imported into Rayyan (QCRI) [18], where duplicates were removed. Two reviewers independently screened titles and abstracts in a blinded manner, followed by full-text eligibility assessment. Disagreements were resolved through discussion and consensus or by involving a third reviewer, if required.

Data Extraction

Data extraction was performed manually using a predefined table. Extracted information included author, study characteristics, sample details, intervention and comparison descriptions, and reported periodontal, esthetic, and functional outcomes. All extracted data were checked twice for accuracy and unit consistency.

Risk of Bias Assessment

The risk of bias for the included studies was assessed according to their respective study designs. As there were no randomized controlled trials included, risk of bias was assessed exclusively using the Risk of Bias in Non-Randomized Studies of Interventions (ROBINS-I) tool [19]. This tool evaluates bias across seven domains: confounding, selection of participants, classification of interventions, deviations from intended interventions, missing data, measurement of outcomes, and selection of the reported result. Each domain was judged as low, moderate, serious, or critical risk of bias, and the overall risk of bias for each study was determined by the highest level of bias identified in any domain. Publication bias could not be formally assessed due to the absence of a meta-analysis.

Effect Measures

Effect measures for continuous variables, including periodontal measurements and esthetic evaluations, were extracted as means and standard deviations, and/or mean differences were reported.​​​​​​​

Synthesis Methods

There was marked variability in outcome definitions and heterogeneity across studies in terms of design, populations, interventions, and outcome metrics, which prevented meta-analysis. A descriptive narrative synthesis was therefore performed. Clinical comparative studies evaluating orthodontic space closure versus definitive prosthetic replacement were synthesized as the primary evidence, with findings grouped into periodontal, esthetic, and functional outcome domains and compared qualitatively between treatment modalities.

Certainty Assessment

The certainty of evidence for each outcome was evaluated using the Grading of Recommendations, Assessment, Development and Evaluation (GRADE) approach [20]. The certainty level was downgraded or upgraded based on five domains: risk of bias, inconsistency, indirectness, imprecision, and publication bias. The overall certainty of evidence was then categorized as high, moderate, low, or very low, according to the GRADE framework.

Results

Study Selection

**Figure 1:**
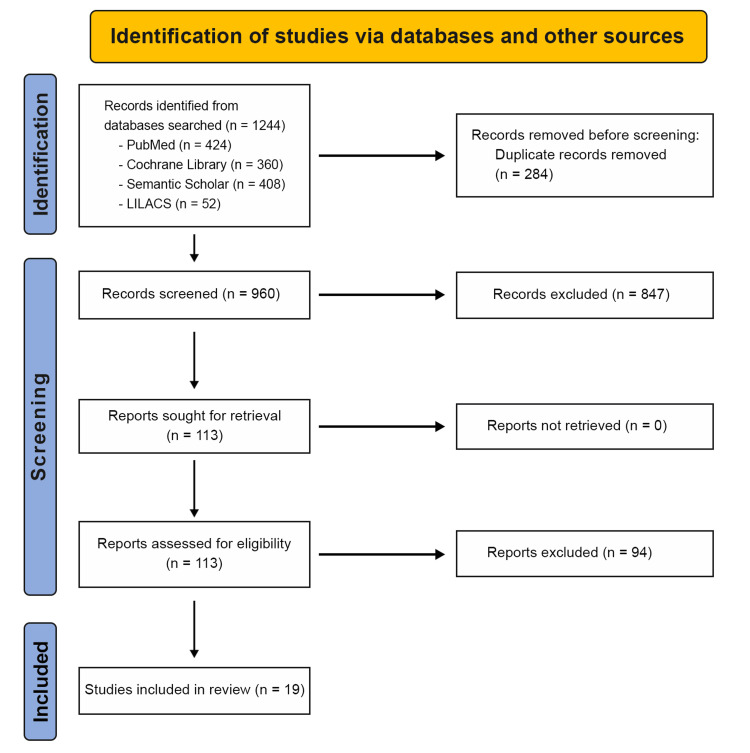
PRISMA Flow Diagram

A total of 1,244 records were identified through database searching (PubMed: 424; Cochrane Library: 360; Semantic Scholar: 408; LILACS: 52). After removal of 284 duplicates, 960 records were screened by titles and abstracts, and 847 were excluded for not meeting the eligibility criteria. The full texts of 113 reports were retrieved and assessed for eligibility. After full-text screening, 94 reports were excluded, and 19 studies were included in the final review. The included studies comprised 18 retrospective studies and one prospective study [21-39]. No randomized controlled trials were included. Figure 1 illustrates the PRISMA flow diagram summarizing the study selection process.

Study Characteristics 

Fifteen studies evaluated esthetic outcomes [21-23,26-34,36-38], seven assessed periodontal outcomes [22,24-26,28,35,39], and six reported occlusal and functional outcomes [22,24,25,27,35,39]. For outcome assessment methods, six studies used clinical examination [22,24-27,35], nine employed photographic evaluation [21,23,31-34,36-38], four utilized questionnaires [22,23,27,39], and five analyzed dental casts or study models [22,24,28-30]. Fourteen studies directly compared orthodontic space closure and prosthetic replacement [21-33,38]. One study compared orthodontic space closure with a control group without prosthetic replacement [35], while three studies evaluated photographic/digitally modified variations of idealized images (e.g., canine width, morphology, shade, and gingival margin levels) [34,36,37]. One study assessed oral-health-related quality of life following orthodontic space closure from an esthetic perspective [39]. Participants’ ages ranged from 14 to 45 years. The key design features, sample characteristics, interventions, assessors, follow-up periods, and outcome assessment methods of the included studies are summarized in Table 3.

**Table 3:** Characteristics of included studies evaluating outcomes of orthodontic space closure and prosthetic replacement for congenitally missing maxillary lateral incisors OSC denotes orthodontic space closure; PR denotes prosthetic replacement. Follow-up refers to the time after completion of orthodontic and/or prosthetic treatment. OSC: orthodontic space closure; PR: prosthetic replacement; CG: control group; FPD: fixed partial denture; RPD: removable partial denture; RBB: resin-bonded bridge (Maryland); TMD: temporomandibular disorders; GZ: gingival zenith; GP: golden proportion; BRG: bridge group; BIG: implant group; NR: not reported; OHRQoL: oral health–related quality of life; F: female; M: male; yrs: years.

Author, Year	Study Design	Aim	Sample Size	Age	Intervention and Comparisons	Assessors	Follow Up Duration (Time after treatment)	Method of Outcome Assessment
De-Marchi et al., 2014 [21]	Retrospective comparative study	Evaluation of smile attractiveness.	68	OSC=14.10-41.10 yrs; PR(Implant)=19.02-45.0; CG=19.07-26.12	OSC=26; PR (Implants)=20; Control group (CG)=22	20 laypersons (10 men, 10 women) and 20 dentists (10 men, 10 women)	6 months	Photographs
Robertsson et al., 2000 [22]	Retrospective study	Evaluation of aesthetics, occlusal function and periodontal health.	50 (F=36, M=14) (Bilateral=39, Unilateral=11)	Mean age= 25.8 yrs.	OSC=30; PR=20	One examiner. Questionnaires completed by subjects	7.1 yrs	Clinical examination, interview, study casts and photographs.
Schneider et al., 2016 [23]	Retrospective study	Evaluation of esthetic outcomes.	9 intraoral photographs	NR	OSC=3, PR=3, CG=3 photographs.	87 orthodontists, 100 general dentists and 100 laypersons.	12-24 months	Photographs, questionnaire.
Nordquist et al., 1975 [24]	Retrospective study	Evaluation of occlusion and periodontal health.	33 patients (66 maxillary quadrants analyzed)	NR	OSC= 39; PR(FPD)=13; PR (RPD)=6, CG=8	NR	2 yrs 4 months to 25 yrs 6 months (mean = 9 years 8 months)	Clinical examination, unmounted study casts, full mouth radiographs.
De-Marchi et al., 2012 [25]	Retrospective study	Evaluation of TMD and periodontal status.	68	OSC= 24.95 yrs; PR (Implants)= 25.12 yrs; CG =21.30 yrs.	OSC=26(9 unilateral, 17 bilateral); PR (Implants) =20 (10 unilateral ,10 bilateral); CG=22	NR	NR	Clinical examination
Hedmo et al., 2024 [26]	Cross-sectional, retrospective study	Evaluation of aesthetic and clinical outcomes.	88 (132 Teeth)	19.5–33.7 yrs	Early cohort = 44; Later Cohort= 44; PR (Implant)= 44; OSC= 44	One examiner	10 years	Clinical examinations
Josefsson & Lindsten, 2019 [27].	Retrospective study	Evaluation of functional and esthetic outcomes.	44 (62 teeth)	PR=24.6-33.7 yrs; OSC=20.5 - 30.7 yrs	PR(Implant)=22; OSC=22	One examiner	At least 5 years	Clinical examinations and questionnaires.
Pini et al., 2012 [28]	Retrospective study	Evaluation of width/length ratio and the gingival zenith(GZ).	52	NR	OSC=18, PR(Implant)=10, CG=24	One examiner	BRG= 5.03 yrs, BIG= 3.08 yrs	Study models made of orthodontic plaster and digital calliper.
Pini et al., 2012 [29]	Retrospective study	Evaluation of the presence of the golden proportion (GP).	73	18-45 yrs	OSC (Unilateral)=10, OSC(Bilateral)=18, PR (Implant unilateral)=10, PR(Bilateral)=10, CG=25	NR	NR	Dental casts and millimeter rule.
Pini et al., 2013 [30]	Retrospective study	Evaluation of aesthetic outcomes.	52	20-26 yrs	OSC=18, PR(Implant)=10, CG=24	One examiner	NR	Dental casts scanned using a 3D scanner .
Moradpoor et al., 2018 [31]	Retrospective study	Evaluation of aesthetic outcomes.	24	26.08 +/- 2.84 yrs	OSC=11, PR(Implants)=13	NR	NR	Photographs (Intraoral)
Qadri et al., 2016 [32]	Retrospective, Cross-sectional, web-based survey	Evaluation of aesthetic outcomes	21 intra-oral photographs	NR	OSC=11, PR (Implant)=10	Five orthodontist, Five restorative dentists	NR	Photographs (Post-treatment, intra oral) and Web-based survey
Hedmo et al., 2022 [33]	Retrospective cross-sectional, quantitative study	Evaluation of aesthetic outcomes	44	PR(Implants)= 24.7-33.8 yrs OSC=20.6- 30.8 yrs.	PR(Implants)=22, OSC=22	Laypeople 20-30 yrs (n=26), laypeople 50-70 yrs (n=26), orthodontists (n=25)	After 5 years after prosthetic therapy	Photographs
Brough et al., 2010 [34]	Retrospective study	Evaluation of aesthetic outcomes	31 images	NR	Variable canine width=6; Crown height and tip morphology=9; Canine gingival margin height=6; Canine shade=10	120 (40 each in orthodontists - 33.9 +/- 7.8 yrs dentists 34.3 +/- 11.2 yrs and laypeople- 36.6 +/- 11.7 yrs)	NR	Photographs
Rosa et al., 2016 [35]	Retrospective cohort study	Evaluation of periodontal and functional health	58	23 yrs 7 months	OSC=26; CG=32	One orthodontist	At least 60 months, mean = 9 years 9 months.	Clinical examination and interviews
Hershaw et al., 2024 [36]	Retrospective study (observational)	Evaluation of aesthetic outcomes	6 Images	NR	A: Ideal gingival margins lateral below central canine level; B: Lateral above central canine below not ideal; C: Lateral below central ideal canine below not ideal; D: All margins level with central ideal for canine only; E: Mixed lateral ideal canine not ideal; F: Both lateral and canine not ideal	120 responders (median age =48 years) 50% male, 81.7% Caucasian , 87% able to provide specific preferences.	NR	Photographs
Rayner et al., 2015 [37]	Prospective , cross-sectional, Non-clinical study	Evaluation of aesthetic outcomes	7 images	NR	1. Control, 2. Extreme bilateral, 3. Extreme unilateral, 4. Average bilateral, 5. Average unilateral, 6. Ideal bilateral, 7. Ideal unilateral	Orthodontists (n=30), dentists (n=30), lay people (n=30), All volunteers over 18 years.	NR	Photographs
Tuero et al., 2025 [38]	Retrospective study	Evaluation of aesthetic outcomes	31 patients	NR	OSC=16, PR (Implants)=15	57 orthodontists, 75 dentists, and 85 laypeople.	NR	Photographs (Intraoral)
Al-Jabrah et al., 2021 [39]	Retrospective study	Evaluation of Oral Health-Related Quality of Life (OHRQoL)	25	Male= 15-21, Females=14-20	Between gender (Male = 10, Female= 15)	NR	NR	Questionnaires

Risk of bias in included studies

**Figure 2:**
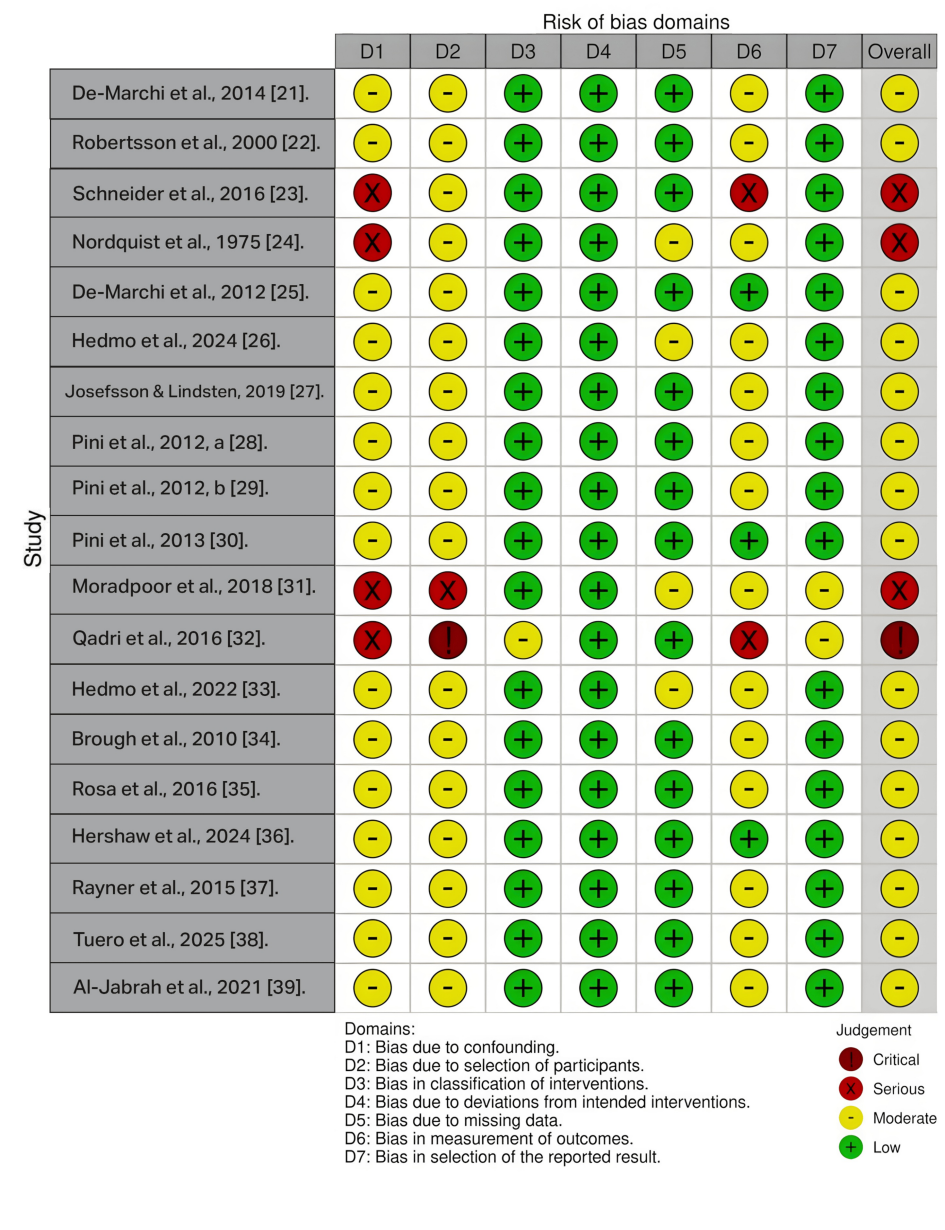
ROBINS-I domain-level risk of bias assessment for included studies Each row represents an included study, and each column represents a ROBINS-I domain (D1–D7). Domain judgments are displayed as low, moderate, serious, or critical risk of bias. ROBINS-I: Risk of bias in non-randomized studies of interventions; D1: bias due to confounding; D2: bias due to selection of participants; D3: bias in classification of interventions; D4: bias due to deviations from intended interventions; D5: bias due to missing data; D6: bias in measurement of outcomes; D7: bias in selection of the reported result.

Overall, the included studies demonstrated a predominantly moderate to serious risk of bias, as assessed using the ROBINS-I tool [19]. The main sources of bias across studies were non-randomized designs, retrospective or cross-sectional methodologies, and non-random participant selection, resulting in a high likelihood of confounding and selection bias across the evidence base [21-39]. Several esthetic perception studies relied on photographic, image-based, or questionnaire-based outcome assessments, which increased susceptibility to subjective outcome measurement, assessor-related confounding, and limited blinding, thereby elevating risk of bias in the measurement domain [21,23,31-34,36-39]. Studies using convenience samples, small sets of pre-selected clinical images, or digitally modified photographs were particularly vulnerable to a serious risk of bias due to non-representative sampling and subjective grading methods [23,32,34,36,37]. In contrast, comparative clinical studies that used standardized periodontal/functional indices, objective cast-based measurements, and/or structured clinical examinations, with more complete follow-up, generally demonstrated a moderate risk of bias, although residual confounding could not be fully excluded [22,24-30,35]. Overall, the lack of randomization and the frequent use of subjective aesthetic outcomes were the most consistent limitations affecting internal validity. The domain-level ROBINS-I judgments for each included study are presented in Figure 2.

**Figure 3:**
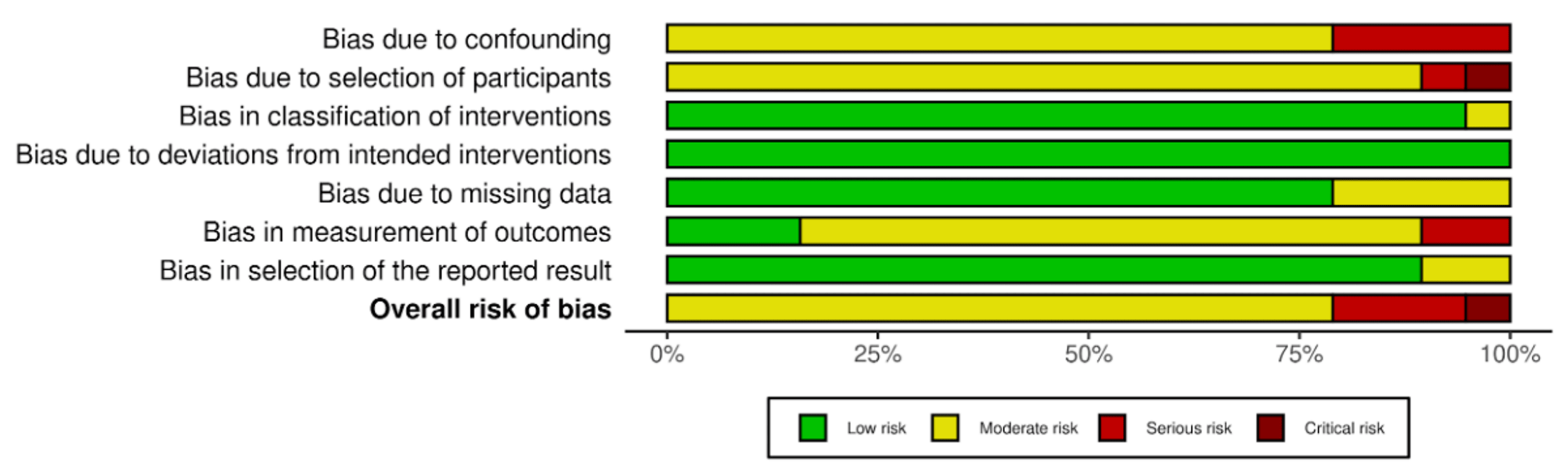
Summary distribution of ROBINS-I risk of bias judgments across domains and overall Stacked bars show the proportion of studies judged as low, moderate, serious, or critical risk of bias for each ROBINS-I domain and for overall risk of bias.

The distribution of ROBINS-I risk-of-bias judgments across domains and the overall risk of bias are displayed in Figure 3.

Results of Individual Studies

The results of individual studies are summarized below according to the primary outcomes assessed, including esthetic, periodontal, occlusal, and patient-reported outcomes.

De Marchi et al. (2014) evaluated smile esthetics using a visual analogue scale (VAS) based on photographic assessment by laypeople and dentists, with ratings ranging from “least attractive” to “most attractive” measured in millimeters. Orthodontic space closure was rated as more esthetically pleasing than prosthetic replacement, and no significant differences were observed between the implant-supported restoration group and the control group [21].

Robertsson et al. (2000) assessed patient satisfaction using the Eastman Esthetic Index questionnaire and categorized responses as “satisfied,” “dissatisfied,” or “no opinion” for tooth shape, color, space distribution, and symmetry. Orthodontic space closure was better accepted than fixed and removable partial dentures. Helkimo’s Dysfunction Index showed no significant difference in the prevalence of temporomandibular dysfunction between groups, while increased plaque accumulation was observed in patients treated with prosthetic replacement [22].

Schneider et al. (2016) evaluated standardized photographs of orthodontic space closure and prosthetic replacement using ratings from orthodontists, general dentists, and laypersons on a five-point scale (1 = best, 5 = worst) across seven bipolar adjective pairs. Implant-supported crowns showed improved esthetic ratings at the 10-year follow-up assessment. Orthodontists and general dentists rated implant therapy and space closure as similarly pleasing, whereas laypersons showed a preference for orthodontic space closure [23].

Nordquist (1975) assessed periodontal and occlusal outcomes using gingival, plaque, irritant, and periodontal indices, with measurements recorded at six sites per tooth: mesiobuccal, buccal, distobuccal, distolingual, lingual, and mesiolingual. The greatest plaque accumulation was observed adjacent to pontics and on the lingual surfaces of maxillary teeth in quadrants restored with removable prostheses. Orthodontic space closure demonstrated healthier periodontal conditions, while fixed partial dentures were more conducive to gingival and periodontal health than removable prostheses. Both groups had similar adequacy of occlusal function, but plaque levels were lower in the space closure group [24].

De Marchi et al. (2012) assessed periodontal status and temporomandibular disorders using a modified Helkimo questionnaire and Research Diagnostic Criteria for TMD. Periodontal conditions were similar across groups, with less gingival recession seen in thick periodontal biotypes. The prosthetic replacement group exhibited higher papilla index values. Both groups did not have any statistically significant differences in terms of signs and symptoms of temporomandibular disorders [25].

Hedmo et al. (2024) reported long-term esthetic outcomes over a 10-year follow-up period. The implant group showed improvements in crown length, bleeding on probing, papilla index, incisor inclination, and overall appearance, while the space closure group showed improvements in crown color and overbite. Additionally, deterioration in crown length was observed over time in orthodontically treated cases [26].

Josefsson et al. (2018) analyzed 12 aesthetic variables. They found better gingival color and crown length outcomes in the orthodontic space closure group, while crown color was rated more favorably in the prosthetic replacement group [27].

Pini et al. (2012) evaluated width/length ratios and gingival zenith using dental casts and calipers. The gingival zenith was assessed in relation to a tangent drawn through the gingival zeniths of the central incisors and canines. No statistically significant differences in width and length ratios were observed between groups. However, orthodontic space closure showed negative gingival zenith values, indicating that substitution of canines for lateral incisors does not reestablish the gingival triangle in accordance with current esthetic recommendations [28].

A second study by Pini et al. (2012) investigated the presence of the golden proportion among the six maxillary anterior teeth using Levin’s grid applied to standardized frontal views. Although the golden proportion has been proposed as an aesthetic guideline, it was not consistently observed in the majority of cases [29].

Pini et al. (2013) evaluated the width/height ratio, the gingival zenith, and Apparent Contact Dimensions (ACD). The orthodontic space closure group had less favorable gingival zenith outcomes, whereas implant-supported restorations showed more favorable gingival zenith but less favorable apparent contact dimensions [30].

Moradpoor et al. (2018) evaluated esthetic outcomes using the Pink Esthetic Score (PES) based on photographic assessment. They found no statistically significant differences between groups, except for the distal papilla, where the mean PES score was significantly lower in the implant group compared with the OSC group [31].

Qadri et al. (2016) assessed esthetic outcomes based on laypeople’s opinions using a web-based survey with a 5-point Likert scale. Ten images that had been selected as most attractive by five orthodontists and five restorative dentists were used for evaluation. Orthodontic space closure was rated as more attractive than prosthetic replacement [32].

Hedmo et al. (2022) assessed esthetic outcomes using a visual analogue scale (VAS) ranging from “very dissatisfied” to “very satisfied,” based on evaluations by laypeople and dental professionals. The color of the gingiva adjacent to implant-supported crowns received lower ratings compared with the space closure group. Laypeople rated both treatment modalities as equally esthetic, whereas orthodontists showed a slight preference for space closure. Interestingly, younger laypeople were more dissatisfied with the tooth color in the space closure canine substitution group [33].

Brough et al. (2010) evaluated the influence of variations in the morphology, size, and shade of maxillary canines on the perception of smile attractiveness using digitally modified images (supplementary esthetic perception evidence). The study demonstrated that changes in canine morphology, width, and color significantly affected perceived smile esthetics [34].

Rosa et al. (2016) evaluated the long-term periodontal and temporomandibular outcomes of orthodontic space closure over a 10-year follow-up period. The control group consisted of orthodontically treated patients without congenitally missing lateral incisors. The study found no evidence of periodontal tissue deterioration associated with orthodontic space closure, including first premolar intrusion and canine extrusion. Additionally, the prevalence of temporomandibular disorders did not increase [35].

Hershaw et al. (2024) evaluated the influence of gingival margin height in cases of maxillary canine substitution using image-based preference ratings (supplementary esthetic perception evidence). Gingival margins with the substituted lateral incisors positioned symmetrically below the central incisors and substituted canines also below this level were ranked most attractive. In contrast, vertical asymmetry of the substituted canine gingival margin was considered the least esthetic [36].

Rayner (2015) evaluated the influence of canine tooth characteristics and symmetry on perceived smile attractiveness in cases of lateral incisor agenesis using standardized images (supplementary esthetic perception evidence). Dental professionals rated smiles with canine substitution as less attractive than an ideal smile unless the substituted canines closely approximated lateral incisors in size, shape, color, and gingival margin. However, laypeople did not perceive canine substitution to be significantly more or less attractive than an ideal smile, regardless of canine characteristics. None of the assessor groups rated unilateral canine substitution as significantly less attractive than bilateral substitution [37].

Tuero et al. (2025) reported that laypeople assigned significantly higher scores than orthodontists and dentists to “bad” images of both orthodontic space closure and space opening with implant placement. Orthodontists and dentists were able to distinguish between good and bad outcomes for both treatment modalities, whereas laypeople showed a statistically significant differentiation only for space opening. Additionally, clear sex-based differences were observed among lay assessors, with men rating unesthetic outcomes more favorably than women [38].

Al Jabrah et al. (2021) evaluated oral-health-related quality of life following orthodontic space closure with canine substitution using OHIP and a modified Eastman Esthetic Index. Most patients reported satisfactory oral health; however, a subset of patients experienced impairment, which was predominantly related to social disability and psychological discomfort. Females reported greater negative psychosocial impact than males, and dissatisfaction patterns differed between sexes for tooth color and shape [39]. The main outcomes, assessment tools, and key inferences reported across all included studies are summarized below in Table 4.

**Table 4:** Summary of individual study findings and outcome assessment instruments OSC denotes orthodontic space closure. PR denotes prosthetic replacement (including implant-supported or tooth-supported prostheses as reported). Values are presented as reported in the original studies. OSC: orthodontic space closure; PR: prosthetic replacement; PRI: prosthetic replacement with implant (if used in the table); CG: control group; FPD: fixed partial denture; RPD: removable partial denture; RBB: resin-bonded bridge (Maryland); VAS: visual analogue scale; PES: pink esthetic score; EEI: Eastman Esthetic Index; OHIP: Oral Health Impact Profile; OHRQoL: oral health–related quality of life; TMD: temporomandibular disorders; TMJ: temporomandibular joint; PD: probing depth; BOP: bleeding on probing; WHR: width/height ratio; GZ: gingival zenith; GP: golden proportion; ACD: apparent contact dimension; GDP: general dental practitioner; NR: not reported; NS: not significant.

Author, Year	Outcome Assessment Instruments	Variables Checked	Outcome	Inference
De-Marchi et al., 2014 [21]	Visual analog scale (VAS)	Attractiveness of smile	Raters’ assessment Laypersons OSC= 41.54±4.63mm PRI= 45.11±13.52mm CG=47.31±10.19mm Dentists OSC= 42.22±16.99mm PRI=43.58±16.28mm CG=49.63±8.46mm	No significant difference found among any of the groups. Patients treated with OSC were more satisfied than patients treated with PRI, even more than the CG.
Robertsson et al., 2000 [22]	Questionnaire for TMD, para functional habits and quality of occlusal contacts. Helkimo’s index of clinical dysfunction Esthetic index.	TMD; Para functional habits; Quality of occlusal contacts; Maximum range of mandibular movements; Deviation of mandibular movements; Deviation of the mandibular path on opening; Temporomandibular joint (TMJ) sound; TMJ locking or luxation; pain during mandibular movements and muscle; TMJ tenderness during palpation; Tooth contacts; Helkimo’s index of clinical dysfunction; Presence of plaque; Bleeding on probing; Pocket depth; Buccal gingival retraction. Examination of prosthesis - Marginal adaptation of the abutments; Size of inter-proximal space; Color.	Esthetic Index: Subjects very or moderately satisfied with appearance of their teeth OSC= 93% PR= 65%. Color of tooth: OSC= 45% satisfied, 55% dissatisfied; PR= 81% satisfied, 19% dissatisfied. Number of locations with plaques: OSC= 1.36±1.58; PR= 2.62±1.51. Number of locations with bleeding after probing: OSC=1.51±1.28; PR=2.61±1.50 Number of locations with retention factors grade b&c: OSC= 0.19±0.68; PR=1.32±1.09	OSC is reasonably stable and better accepted by the patients than PR (FPD,RPD). Single implants not included in the study.
Schneider et al., 2016 [23]	Questionnaire (Bashara and Jakobson)	Esthetic appeal	Orthodontists CG = 12.84 ± 0.43 PRI = 15.90 ± 0.56 OSC = 17.25 ± 0.51 No significant preference between OSC and PRI Dentists CG = 13.35 ± 0.49 OSC = 15.58 ± 0.59 PRI = 15.12 ± 10.61 No statistically significant difference between OSC and PRI Laypersons OSC = 13.97 ± 0.53 CG = 14.71 ± 0.55 PRI = 16.19 ± 0.61	Esthetic outcomes of implant-borne crowns replacing missing maxillary lateral incisors more appealing, but preferences can vary between dental professionals and laypersons. Laypersons preferred OSC over PRI; implant-borne crowns showed the greatest statistically significant improvement across all respondent groups.
Nordquist et al., 1975 [24}	Loe and Silness gingival index, Loe’s retention index, Loe’s plaque index, Green’s oral hygiene index, Periodontal index.	Gingival color change, edema, bleeding on probing, tissue contour, and ulceration, presence and severity of irritants other than plaque, pocket depth, occlusal contacts.	Plaque accumulation: Highest adjacent to lateral incisor pontics and lingual surfaces with RPDs (t = 6.66 anterior, t = 5.08 posterior) Mechanical irritants: Greatest in FPD cases (t = 5.72) Gingival index: Significantly higher in quadrants with fixed or removable prostheses (F = 27.25) Pocket depth: Greater with fixed prostheses, especially anterior teeth (t = 4.43) Occlusal pattern: Group function in 63/66 quadrants; cuspid rise in 3/66 quadrants Premature contacts: Teeth with >1 premature contact showed greater pocket depth (t = 8.64) OSC vs open space: No significant differences in premature centric/excursive contacts No significant between-group differences in periodontal index	Orthodontic space closure provides healthier periodontal conditions and comparable occlusal function to prosthetic replacement, making it the preferred option for managing congenitally missing maxillary lateral incisors when feasible.
De-Marchi et al., 2012 [25]	Modified Helkimo questionnaire & Research Diagnostic Criteria for TMD.	Plaque index, bleeding upon probing, probing depth, Papilla index, abfraction lesions, periodontal bio type, gingival recession, TMD.	Plaque index: OSC= 61±13%, PRI= 52±11% (NS) Bleeding index: OSC= 11±18%, PRI= 7±6% (NS) Probing depth >3mm; OSC= 15 faces (1%) PRI= 25 faces (1.7%). Periodontal biotype: Thick biotype associated with SOI; thin biotype associated with SCR and CG (Fisher’s exact test). Medial papilla: Statistically significant differences among SCR, SOI, and CG for maxillary right and left lateral incisors. Parafunction: Reported by 3 SCR patients and 5 SOI patients. TMD: No statistically significant intergroup differences in signs or symptoms.	Orthodontic space closure provides periodontal and occlusal outcomes comparable or superior to prosthetic replacement, with reduced plaque accumulation and no compromise in function.
Hedmo et al., 2024 [26]	-	1. Aesthetics of the tooth replacing missing maxillary lateral incisors: a. Crown color, b. Color of adjacent gingiva, c. Crown length 2. Gingival conditions of tooth replacing missing maxillary lateral incisor; a. Visible implant, b. Gingival. Recession, c. Bleeding on probing, d. Papillae. 3. Occlusal morphology and extra oral assessment: a. Sagittal dental relationship, b. Space conditions, c. Overjet, d. Overbite, e. Inclination of maxillary incisors, f. Midline in upper arch, g. Lip closure, h. Overall appearance while smiling.	Aesthetics Implant therapy: Non acceptable gingival color ( EC= 60.5%, LC= 73.5%, NS), Abnormal crown length (EC= 60.5%, LC=29.5) Space closure: Non-acceptable crown color (EC=20.5%, LC=0), Abnormal crown length (EC=14.5%, LC=47%) Gingival conditions: Implant therapy: Bleeding on probing (EC=85.5%, LC=53), Papillae defect (EC= 68%, LC= 38%) Space closure: Bleeding on probing (EC=35.5%, LC=5.5%) Occlusal morphology and extra oral assessment Implant therapy; Proclination of maxillary incisors (EC=45.5%, LC= 0), Strained lip closure (EC=22.5%, LC=0%)	Compared with baseline, space-closure cohorts showed improvement in crown color and overbite over time, while crown length and bleeding on probing worsened.
Josefsson et al., 2019 [27]	-	1. The aesthetics of the implant supported the crown and the canine replacing the lateral incisor: a. Color of the adjacent gingiva, b. Crown color, c. Crown length. 2. Gingival conditions of the implant supported crown and canine replacing the lateral incisor: a. Visible screws, b. Bucccal gingival recession, c. Bleeding on probing, d. Papilla formation. 3. Occlusal morphology and extra oral assessment: a. Sagittal dental relationship, b. Space condition, c. Overjet, d. Overbite, e. Inclination of maxillary incisors, f. Midline in upper jaw, g. Lip closure, h. Appearance when smiling, i. Harmony of the study tooth to the rest of the dentition. Patients and examiners were asked if they were satisfied with the aesthetic result and appearance of the anterior teeth.	Esthetic and periodontal findings (PRI vs OSC): Gingival color (non-acceptable): PRI 61%, OSC 9% Crown color (non-acceptable): PRI 0%, OSC 21% Abnormal crown length: PRI 61%, OSC 15% Buccal gingival retraction: NS Bleeding on probing: NS Papilla defect: NS Occlusal / positional variables: Midline deviation (maxilla): NS Anterior maxillary spacing: NS Tooth harmony with adjacent teeth: NS Proclination of maxillary incisors: PRI 32%, OSC 4% Strained lip closure: PRI 23%, OSC 0% Non-acceptable smile appearance: PRI 32%, OSC 9% Overall esthetic satisfaction – Patients: PRI: Satisfied 68%, Acceptable 27%, Not acceptable 5% OSC: Satisfied 59%, Acceptable 41%, Not acceptable 0% Overall esthetic satisfaction – Examiners: PRI: Satisfied 23%, Acceptable 59%, Not acceptable 18% OSC: Satisfied 41%, Acceptable 45%, Not acceptable 14% Between-group comparison: No significant difference in overall patient or examiner satisfaction with maxillary anterior teeth.	More variables showed significantly better outcomes in the space closure group than in the implant group; therefore, space closure should be recommended when feasible, using a coordinated multidisciplinary treatment approach.
Pini et al., 2012 [28]	-	Width/Length ratios	Width/length ratio of lateral incisors: PRI showed the lowest mean values (0.72 right, 0.72 left). Statistically significant differences were observed for the right lateral incisor (PRI vs CG) and for the canine (OSC vs CG). Gingival zenith (GZ) ratios: OSC showed the greatest discrepancy (0.50 right, 0.48 left). PRI (0.95 right, 0.98 left) and CG (0.98 right, 0.80 left) showed similar values with no statistically significant difference (p > 0.05). No significant differences were observed between right and left sides.	No statistically significant differences in width–length ratio were observed among patients with bilateral lateral incisor agenesis treated with recontouring, implants, or controls; however, median lateral incisor values were lower in the implant group. Gingival zenith values differed most in the recontouring group, showing negative values and indicating inadequate gingival triangle formation.
Pini et al., 2012 [29]	-	Golden proportion	Golden Proportion (GP) and Width/Height Ratios GP incidence: Significantly different between groups; more frequent between central–lateral than lateral–canine teeth Groups with higher GP presence: OSC, PRI(Unilateral), CG Maxillary agenesis cases: GP absent in the majority, irrespective of treatment method Mean lateral incisor W/H ratio: 0.75–0.90	Golden proportion was inconsistently present, occurred more frequently between central and lateral incisors, was less common in treated agenesis cases irrespective of treatment modality, and lateral incisor proportions showed wide variability.
Pini et al., 2013 [30]	-	Width/Height Ratio (WHR), Gingival zenith (GZ), Apparent contact dimension (ACD)	WHR No significant differences among groups OSC and PRI lower than CG Gingival zenith OSC consistently lower than PRI and CG Inverted gingival triangle predominant in PRI ACD PRI highest OSC intermediate CG lowest	Patients treated with space closure and recontouring were closest to controls for width/height ratio and apparent contact dimension but showed the greatest deviation in gingival zenith, indicating failure to reestablish the gingival triangle, while implant-supported prostheses exhibited smaller width/height ratios, more positive yet apically positioned gingival zeniths, and greater divergence in apparent contact dimensions compared with controls.
Moradpoor et al., 2018 [31]	Pink esthetic score (PES)	Mesial papilla, Distal papilla, Curvature of facial mucosa, Level of facial mucosa, Root convexity, soft tissue color and texture	Distal Papilla: PRI=1.08 +/- 0.49 OSC= 1.73 +/- 0.47 (p= 0.013)	No significant esthetic differences were observed between space opening with implant placement and space closure with canine reshaping based on PES, except for distal papilla scores, which were significantly lower in the implant group.
Qadri et al., 2016 [32]	Five-point Likert scale	Part 1 (attractiveness) - 10 single images that were ranked highest for attractiveness by clinicians, placed in a random order. Respondents were asked to assess each image on a 5-point Likert scale. Part 2 (Preference) Consisting of four paired images per screen that were ranked most attractive by the clinicians. The highest ranked OSC image was placed with the highest ranked PR image, followed by the next ranked images, etc.	OSC Attractive/very attractive = 45.7% Unattractive/very unattractive = 20.1% PR Attractive/very attractive = 40.5% Unattractive/very unattractive = 29.3%	Laypeople rated orthodontic space closure and camouflage as equally or more attractive than space opening with prosthetic replacement, with a majority preferring space closure over prosthetic replacement.
Hedmo et al., 2022 [33]	Horizontal Visual Analogue scale (VAS: 100)	1.The aesthetic of the dentition. 2. The aesthetics of the dentition compared to other people in general, 3. The shape of the replacing tooth, 4. The color of the replacing tooth, 5. Color of the gingiva adjacent to the replacing tooth, 6. The midline.	The aesthetics of the dentition; Orthodontist - OSC - 65-77%, PRI - 56-69% Color of the gingiva adjacent to the replacing tooth Orthodontists OSC=88-92% PRI=31-47% Young laypeople OSC=55-77% PRI=29-44% Older laypeople OSC=62-81% PRI=33-53%	Gingival color adjacent to implant-supported crowns was rated less aesthetic than space closure; laypeople rated both treatments similarly, while orthodontists showed a slight preference for space closure, and younger laypeople were more dissatisfied with tooth color in the space-closure group.
Brough et al., 2010 [34]	-	1. Canine width, 2. Canine height and tip morphology, 3. Canine shade, 4. Gingival margin height.	Attractiveness ratings OSC attractive/very attractive 45.7% PR attractive/very attractive 40.5% OSC unattractive/very unattractive 20.1% PR unattractive/very unattractive 29.3% Canine width preference 1.5 mm narrower canine attractive 50% Original canine width next preferred 3 mm narrower width next preferred 3 mm wider than original least attractive 71.67% Canine shade preference 10× brighter canines most attractive 23.33% 5× brighter next preferred 15× brighter next preferred Original shade next preferred 20× darker canines least attractive 78.33%	Canine morphology significantly influences smile esthetics, with narrower canines perceived as most attractive and wide or dark canines consistently rated as least attractive by orthodontists, dentists, and laypeople.
Rosa et al., 2016 [35]	-	1. Probing depth (PD), 2. Bleeding on probing, 3. Labial gingival recession and increased tooth mobility, 4. Occlusal functional patterns.	Periodontal and occlusal outcomes OSC PD >4 mm: 0.5% sites PD = 4 mm: 2.4% sites (mainly posterior) Mobility grade I: 4.4% teeth Group function: 92.3% Canine guidance: 7.7% CG PD >4 mm: 0.7% sites PD =4 mm: 3.9% sites Canine guidance: 32% Between groups Bleeding on probing: NS Tooth mobility: NS Other periodontal parameters: NS	Patients treated with orthodontic space closure demonstrate long-term periodontal health comparable to individuals with intact dentitions, with no increased risk of periodontal breakdown or occlusal dysfunction.
Hershaw et al., 2024 [36]	-	A: Lateral below central; canine level (ideal), B: Lateral above central; canine below (not ideal), C: Lateral below central (ideal); canine below (not ideal), D: All margins level with central (ideal for canine only), E: Mixed sides; lateral ideal, canine not ideal, F: Mixed sides; lateral above central (not ideal), canine below (not ideal)	Image preference ranking Image C = 29.8% Image B = 20.2% Image D = 18.3% Image F = 15.8% Image A = 8.7% Image E = 4.9%	Gingival margins with laterals positioned symmetrically below the central incisors were rated most esthetic, whereas vertical asymmetry of substituted canines was perceived as the least esthetic, while asymmetry of substituted lateral incisors had minimal impact on attractiveness.
Rayner et al., 2015 [37]	Visual analogue scale (VAS 100mm)	The canine size, shape, color and gingival margin. The smiles.	Orthodontists Highest scores for unilateral canine substitution with ideal canine form GDPs and laypersons Highest scores for bilateral canine substitution with ideal canine form Dental professionals Canine substitution rated less attractive than ideal smiles unless size, shape, color, and gingival margins approximated lateral incisors Laypersons No significant difference between canine substitution and ideal smiles Group comparison Dental professionals more sensitive to esthetic deviations than laypersons Laterality No significant difference between unilateral and bilateral canine substitution across groups	Orthodontists and GDPs rated canine substitution as less attractive than an ideal smile unless the substituted canines closely matched ideal size, shape, color, and gingival margins, while laypeople perceived minimal esthetic deviation; all groups found no significant difference between unilateral and bilateral canine substitution, and professionals were more sensitive to deviations from ideal smile esthetics than laypeople.
Tuero et al., 2025 [38]	Visual analog scale (VAS)	Smile symmetry and harmony, Camouflage of canines moved into the lateral incisor position, considering shape, size, and color; Aesthetic outcomes of implants placed in the lateral incisor position, considering position, shape, crown color, and gingival color.	Laypersons rated “bad” images significantly higher than orthodontists and dentists for both OSC and PRI. Orthodontists and dentists showed significant differences between “good” and “bad” images for both treatments. Laypersons showed significant differences only for space opening. Significant sex differences were observed among laypersons when rating “bad” cases for both treatments.	Esthetic evaluations differed significantly among assessor groups, with laypersons rating poor outcomes more favorably than professionals, while space opening with implants was consistently judged less esthetic than space closure, particularly for unfavorable cases, and sex-based differences were noted among lay observers.
Al-Jabrah et al., 2023 [39]	Eastman Esthetic Index (EEI) questionnaires and five-point Likert scale.	Functional limitation, pain, psychological discomfort, physical disability, psychological disability, social disability and handicap.	Females OHIP = higher across all domains EEI = lower than males Oral health impact = 33.3% Dissatisfied with shape = higher (p = 0.022) Main impact domains = psychological discomfort and disability Males OHIP = lower than females EEI = higher than females Oral health satisfactory = higher proportion Oral health impact = 20% (p = 0.014) Dissatisfied with color = higher (p = 0.049) Main impact domain = social disability related to tooth color (p = 0.00078)	Social disability was the most affected OHRQoL domain. Females reported significantly greater psychological and social impacts and dissatisfaction with tooth shape and size, while males were more dissatisfied with tooth color. Approximately one-fourth of patients reported impaired OHRQoL, with impacts predominantly related to social disability and psychological discomfort.

Certainty of Evidence

Overall, the certainty of evidence across the included studies was rated as low. This rating was primarily due to the observational and non-randomized study designs, which downgraded the risk of bias across all studies. Additional downgrading occurred due to imprecision (small sample sizes and limited comparative studies) and indirectness, particularly in image-based and questionnaire-based esthetic assessments [21-23,27,31-34,36-39]. Despite these limitations, the evidence remained clinically relevant to the management of congenitally missing maxillary lateral incisors and showed generally consistent findings across esthetic, periodontal, occlusal or functional, and quality-of-life outcomes. The certainty of evidence for each included study, as assessed using the GRADE domains of risk of bias, inconsistency, indirectness, imprecision, and publication bias, is summarized in Table 5.

**Table 5:** GRADE certainty assessment for included studies GRADE: Grading of Recommendations, Assessment, Development and Evaluation; ROB: risk of bias. In accordance with the GRADE system, “⊕” indicates no serious concern for that domain, whereas “◯” indicates downgrading due to methodological limitations. Overall certainty is summarized as combinations of symbols (for example: ⊕⊕◯◯ = Low; ⊕◯◯◯ = Very low).

Author, Year	Risk of Bias	Inconsistency	Indirectness	Imprecision	Publication Bias	Overall
De-Marchi et al., 2014 [21]	◯ Serious	⊕ Not serious	⊕ Not serious	◯ Serious	Unclear	⊕⊕◯◯ LOW
Robertsson et al., 2000 [22]	◯ Serious	⊕ Not serious	⊕ Not serious	◯ Serious	Unclear	⊕⊕◯◯ LOW
Schneider et al., 2016 [23]	◯ Serious	◯ Serious	⊕ Not serious	◯ Serious	Unclear	⊕⊕◯◯ LOW
Nordquist et al., 1975 [24]	◯ Serious	⊕ Not serious	⊕ Not serious	◯ Serious	Unclear	⊕⊕◯◯ LOW
De-Marchi et al., 2012 [25]	◯ Serious	⊕ Not serious	⊕ Not serious	◯ Serious	Unclear	⊕⊕◯◯ LOW
Hedmo et al., 2024 [26]	◯ Serious	⊕ Not serious	⊕ Not serious	◯ Serious	Unclear	⊕⊕◯◯ LOW
Josefsson et al., 2018 [27]	◯ Serious	⊕ Not serious	⊕ Not serious	◯ Serious	Unclear	⊕⊕◯◯ LOW
Pini et al., 2012 [28]	◯ Serious	⊕ Not serious	⊕ Not serious	◯ Serious	Unclear	⊕⊕◯◯ LOW
Pini et al., 2012 [29]	◯ Serious	⊕ Not serious	⊕ Not serious	◯ Serious	Unclear	⊕⊕◯◯ LOW
Pini et al., 2013 [30]	◯ Serious	⊕ Not serious	⊕ Not serious	◯ Serious	Unclear	⊕⊕◯◯ LOW
Moradpoor et al., 2018 [31]	◯ Serious	⊕ Not serious	⊕ Not serious	◯ Serious	Unclear	⊕⊕◯◯ LOW
Qadri et al., 2016 [32]	◯ Serious	⊕ Not serious	◯ Serious	⊕ Not serious	Unclear	⊕⊕◯◯ LOW
Hedmo et al., 2022 [33]	◯ Serious	⊕ Not serious	⊕ Not serious	◯ Serious	Unclear	⊕⊕◯◯ LOW
Brough et al., 2010 [34]	◯ Serious	⊕ Not serious	◯ Serious	⊕ Not serious	Unclear	⊕⊕◯◯ LOW
Rosa et al., 2016 [35]	◯ Serious	⊕ Not serious	⊕ Not serious	◯ Serious	Unclear	⊕⊕◯◯ LOW
Hershaw et al., 2024 [36]	◯ Serious	⊕ Not serious	⊕ Not serious	◯ Serious	Unclear	⊕⊕◯◯ LOW
Rayner et al., 2015 [37]	◯ Serious	⊕ Not serious	◯ Serious	⊕ Not serious	Unclear	⊕⊕◯◯ LOW
Tuero et al., 2025 [38]	◯ Serious	⊕ Not serious	◯ Serious	⊕ Not serious	Unclear	⊕⊕◯◯ LOW
Al-Jabrah et al., 2021 [39]	◯ Serious	⊕ Not serious	⊕ Not serious	◯ Serious	Unclear	⊕⊕◯◯ LOW

Discussion

Maxillary lateral incisor agenesis (MLIA), a commonly encountered condition in dental practice [1-7,40], is typically managed using either orthodontic space closure with canine substitution or space opening followed by prosthetic replacement [11-16,41]. This systematic review synthesized the available evidence comparing orthodontic space closure and prosthetic replacement for the management of MLIA. Given the absence of randomized controlled trials and the predominance of observational and esthetic perception studies, the findings should be interpreted while keeping in mind study design limitations and heterogeneity in outcome assessment. The primary objective of this review was to evaluate and compare esthetic, periodontal, and occlusal or functional outcomes associated with orthodontic space closure and prosthetic replacement. The secondary objectives included assessing patient-reported outcomes, professional and lay perceptions of esthetics, and the influence of canine substitution characteristics on perceived smile attractiveness. Accordingly, the discussion is structured around esthetic outcomes, periodontal health, and occlusal and functional considerations, with emphasis on the clinical relevance of these findings for treatment planning.

Esthetic Outcomes

Out of the included studies, 15 studies evaluated esthetic outcomes following management of maxillary lateral incisor agenesis. Overall, laypeople were more satisfied with orthodontic space closure (OSC) compared with prosthetic replacement with implants (PRI), whereas dental professionals were more critical. Several authors highlighted that long-term implant-related changes, such as infraocclusion, papilla loss, gingival discoloration, and restorative aging, can negatively influence smile attractiveness over time [14,26,27,33]. Although Schneider et al. reported implant-borne crowns to be more esthetically appealing in contrast to earlier findings by Armbruster et al. (2005), this difference was attributed to advancements in prosthetic materials and techniques over time [23,42,43]. Across studies, the implant group had a less favorable gingival color, while the OSC group had a less favorable crown color due to limitations in reproducing the ideal lateral incisor shade with substituted canines [27,33]. Two studies additionally reported increased incisor inclination in the PRI group [26,27]. Even when crown color was acceptable in OSC, gingival zenith discrepancies were commonly observed and often resulted in a negative gingival triangle formation. In contrast, papilla deficiencies were more prominent around implant restorations [26-28,31]. Several studies consistently demonstrated that laypeople often preferred or equally accepted OSC compared with PR, even when canine substitution deviated from ideal morphology [21-23,32,38]. In contrast, orthodontists and general dentists rated canine substitution as less attractive unless the substituted canine closely approximated a lateral incisor in size, shape, color, and gingival margin position, reflecting greater professional sensitivity to esthetic deviations. Image-based and digitally modified studies further emphasized the influence of canine morphology, width, shade, and gingival margin symmetry on smile perception [34,36,37]. Narrower and lighter canines were generally preferred, while wider or darker canines negatively affected attractiveness [34]. Moreover, studies using objective esthetic indices, including the Pink Esthetic Score, gingival zenith, and width-to-height ratios, demonstrated minimal overall differences between OSC and PR, with isolated differences primarily related to papilla and gingival architecture [28,30,31]. Classical esthetic proportions, such as the golden proportion, were inconsistently present across treatment modalities; this suggests that strict adherence to idealized esthetic norms may not be essential for acceptable outcomes, particularly from the perspective of the patient and layperson [29]. These findings align with Guo et al., who similarly reported a preference for OSC over implant-based replacement among lay observers [44].

Periodontal Outcomes

Across the reviewed literature, patients treated with orthodontic space closure achieved generally favorable periodontal outcomes, comparable to those observed in individuals with natural dentition [22,24,25]. Long-term follow-up studies consistently demonstrated that space closure did not increase the risk of periodontal tissue deterioration, gingival recession, or attachment loss, even when treatment involved premolar intrusion and canine extrusion [35]. In contrast, prosthetic replacement, particularly fixed and removable partial dentures, was more frequently associated with increased plaque accumulation, mechanical irritants, and localized periodontal changes [24]. While implant-supported restorations generally demonstrated acceptable periodontal parameters, papillary alterations and gingival color discrepancies were occasionally reported, which may influence overall esthetics [26,27,33]. Chang et al. also reported similar observations of soft tissue changes around prosthetic restorations [45]. Periodontal biotype emerged as an important modifying factor, with thick biotypes showing reduced gingival recession regardless of treatment modality [25]. Nevertheless, some implant-treated groups demonstrated greater papilla-related changes, suggesting potential limitations in achieving optimal soft-tissue architecture [26,31]. Importantly, several studies reported no statistically significant differences in overall periodontal indices between treatment approaches, indicating the periodontal safety of both orthodontic space closure and prosthetic replacement when appropriately planned and maintained [27].

Occlusal and Functional Outcomes

Occlusal and functional outcomes were generally comparable between patients treated with space closure and those managed with prosthetic replacement or intact dentitions [22,24-27,35]. The majority of patients exhibited group function during lateral excursions, and there were no clinically relevant differences in occlusal adequacy between open and closed space treatments. In addition, studies evaluating temporomandibular disorders using standardized diagnostic tools found no increased prevalence of signs or symptoms associated with orthodontic space closure or prosthetic replacement [22,25]. Long-term follow-up data further confirmed that orthodontic tooth movements used in space closure did not adversely affect occlusal function or joint health [26,35]. Overall, the evidence does not support the need to achieve a universal Class I canine relationship or specific occlusal scheme in patients with congenitally missing lateral incisors. As long as the appropriate finishing and occlusal equilibration are performed, functional balance appears to be maintained across treatment modalities. This finding is consistent with the observations of Droukas et al. and Pilley et al.; both independently reported no significant differences in the prevalence of temporomandibular disorders between space closure and prosthetic replacement groups [46,47].

Summary Interpretation

Overall, the current body of evidence indicates that orthodontic space closure can produce esthetic outcomes that are generally acceptable to patients and lay observers, while providing periodontal and functional results that are comparable to, and in some studies, more favorable than, those reported for prosthetic replacement. Although dental professionals often judge canine substitution more critically, particularly when canine morphology, shade, or gingival architecture deviates from ideal lateral incisor characteristics, this professional concern is not consistently mirrored in patient-reported satisfaction. In practical terms, the findings suggest that carefully finished space closure, which includes reshaping and soft-tissue considerations, is a predictable option that balances esthetic acceptability with periodontal stability and functional safety.

Limitations

Several limitations should be considered when interpreting these findings. First, this review was not prospectively registered (e.g., on PROSPERO or an equivalent registry), which increases the risk of selective methodological decisions and reduces transparency regarding a priori planning. Second, the evidence base is dominated by retrospective and cross-sectional designs, with minimal prospective data, making the results vulnerable to selection bias, confounding, and limited control of baseline differences between treatment groups. Third, there was substantial heterogeneity in outcome definitions and assessment methods, spanning clinical periodontal indices, cast-based measurements, varied esthetic indices, questionnaires, and photographic or digitally manipulated image ratings; this prevented quantitative pooling and meta-analysis. Fourth, several esthetic perception studies relied on a small number of clinical cases or pre-selected images; while rater numbers were sometimes large, the limited case spectrum restricts external validity and generalizability to routine clinical presentations. Fifth, observer-related bias remains a major concern because esthetic judgments differed systematically between professionals and laypersons, and blinding or calibration was inconsistently reported. Finally, variations in participant age, timing of assessment after treatment, orthodontic finishing protocols, prosthetic designs and materials, and follow-up durations limit direct comparability across studies and prevent firm conclusions about the long-term superiority of either approach.

## Conclusions

Based on the available evidence, both orthodontic space closure and prosthetic replacement can provide acceptable periodontal, esthetic, and functional outcomes in the management of congenitally missing maxillary lateral incisors. However, orthodontic space closure generally demonstrates more favorable periodontal conditions and esthetic results that are at least comparable to prosthetic replacement, particularly from the perspective of patients and lay observers, while also avoiding long-term prosthesis-related risks such as soft-tissue remodeling, infraocclusion, and restorative aging. Occlusal and functional outcomes appear broadly similar between the two modalities, with no consistent evidence of increased temporomandibular dysfunction when treatment is appropriately finished and maintained. Given the predominantly observational and heterogeneous evidence base, these conclusions should be interpreted with caution. When clinically feasible, orthodontic space closure may be considered a preferred option, but final treatment selection should be individualized according to skeletal and occlusal characteristics, periodontal biotype and soft-tissue profile, patient age and growth status, esthetic expectations, and the anticipated long-term maintenance burden, ideally within a coordinated multidisciplinary treatment plan.
